# Integrative Subtype Discovery in Glioblastoma Using iCluster

**DOI:** 10.1371/journal.pone.0035236

**Published:** 2012-04-23

**Authors:** Ronglai Shen, Qianxing Mo, Nikolaus Schultz, Venkatraman E. Seshan, Adam B. Olshen, Jason Huse, Marc Ladanyi, Chris Sander

**Affiliations:** 1 Department of Epidemiology and Biostatistics, Memorial Sloan-Kettering Cancer Center, New York, New York, United States of America; 2 Division of Biostatistics, Dan L. Duncan Cancer Center, Baylor College of Medicine, Houston, Texas, United States of America; 3 Computational Biology Program, Memorial Sloan-Kettering Cancer Center, New York, New York, United States of America; 4 Department of Epidemiology and Biostatistics, University of California San Francisco, San Francisco, California, United States of America; 5 Department of Pathology and Human Oncology and Pathogenesis Program, Memorial Sloan-Kettering Cancer Center, New York, New York, United States of America; Dana-Farber Cancer Institute, United States of America

## Abstract

Large-scale cancer genome projects, such as the Cancer Genome Atlas (TCGA) project, are comprehensive molecular characterization efforts to accelerate our understanding of cancer biology and the discovery of new therapeutic targets. The accumulating wealth of multidimensional data provides a new paradigm for important research problems including cancer subtype discovery. The current standard approach relies on separate clustering analyses followed by manual integration. Results can be highly data type dependent, restricting the ability to discover new insights from multidimensional data. In this study, we present an integrative subtype analysis of the TCGA glioblastoma (GBM) data set. Our analysis revealed new insights through integrated subtype characterization. We found three distinct integrated tumor subtypes. Subtype 1 lacks the classical GBM events of chr 7 gain and chr 10 loss. This subclass is enriched for the G-CIMP phenotype and shows hypermethylation of genes involved in brain development and neuronal differentiation. The tumors in this subclass display a Proneural expression profile. Subtype 2 is characterized by a near complete association with EGFR amplification, overrepresentation of promoter methylation of homeobox and G-protein signaling genes, and a Classical expression profile. Subtype 3 is characterized by NF1 and PTEN alterations and exhibits a Mesenchymal-like expression profile. The data analysis workflow we propose provides a unified and computationally scalable framework to harness the full potential of large-scale integrated cancer genomic data for integrative subtype discovery.

## Introduction

Cancer genomes harbor a plethora of somatically acquired aberrations. DNA copy number aberrations are key characteristics of cancer, contributing to genomic instability and gene deregulation [1], [2] such as oncogene activation by gene amplification or tumor suppressor loss as a result of gene deletion. Epigenetic aberrations such as DNA methylation are also widespread in the cancer genome [3]. Genome-wide hypomethylation causes genome instability, and hypermethylation of CpG islands has been associated with inactivation of tumor suppressor genes. Many of these genomic changes in the DNA may affect the expression level of messenger RNA (mRNA) as well as non-coding microRNAs, alter the function of the gene product, and ultimately lead to abnormal cellular and biological functions that contribute to tumorigenesis.

Large-scale cancer genome projects including the Cancer Genome Atlas (TCGA) and the International Cancer Genome Consortium (ICGC) are generating an unprecedented amount of multidimensional data using high resolution microarray and next-generation sequencing platforms. With the accumulating wealth of multidimensional data, there is a great need for methods geared toward integrative analysis of multiple genomic data sources. New methods for this type of analysis have been developed. Several recent studies consider pathway and network analysis using multidimensional data [4], [5]. A number of others [6]–[11] suggest using canonical correlation analysis (CCA) to quantify the correlation between two data sets (e.g., gene expression and copy number data). None of these methods are specifically designed for tumor subtype analysis in an integrative fashion.

The clinical and therapeutic implications for many existing molecular subtypes of cancer remain largely unknown. Prioritization of candidate markers relies to a great extent on existing knowledge of cancer biology. To that end, integrating multiple data types (e.g., copy number and gene expression) can provide key information to pinpoint the genomic alterations that characterize disease subtypes of biological and clinical importance (e.g., *HER2* oncogene activation through concordant DNA amplification and mRNA overexpression). Individually, none of the data types completely capture the complexity of the cancer genome or precisely pinpoint the cancer driving mechanism. Collectively, however, integrative genomic studies provide a new paradigm for the discovery of novel cancer subtypes and associated cancer genes.

The current standard analysis involves separate clustering of different genomic data types followed by a manual integration of the cluster assignments. Results can be highly data type dependent, restricting the ability to discover additional insights from multidimensional data. Correlation between data types cannot be utilized in a separate clustering approach, causing substantial loss of information. Another challenge with standard clustering algorithms is that feature selection is not part of the clustering procedure. Typically, all features that pass some initial variance filtering step are included for clustering. The result can be high variable due to noise accumulation in estimating the population cluster centroids in high dimensional feature space. An example can be seen in Supplementary Figure S1E. As a result, sparse clustering has generated much attention in recent statistical literature [12]–[16], assuming a small fraction of the features are directly relevant for class discovery. Statistical inference in high dimensional data setting becomes more reliable with the sparsity assumption. Correct selection of the class-discriminant features crucially affects model interpretation, statistical accuracy, and computational complexity. Yet most widely applied clustering methods are decoupled from the procedure of selecting cluster-discriminant features.

In a previous publication [17], we introduced an integrative clustering method called iCluster based on a Gaussian latent variable model with lasso [18] type penalty terms to induce sparsity in the coefficient matrices toward feature selection. In this paper, we present an integrative analysis workflow using iCluster and demonstrate its utility in defining molecular subtypes of glioblastoma multiforme (GBM) by simultaneously clustering genome-wide DNA copy number, methylation, and gene expression data derived from the TCGA GBM samples. We implemented a modified algorithm using a variance weighted penalty term that is proportional to the error variance associated with each feature. As a result, coefficients will be more heavily penalized for features demonstrating high variance. We discuss the details of the weighted shrinkage estimates in the Methods Section.

## Results

### A unified framework for clustering, data integration, dimension reduction, and feature selection

Supplementary Figure S1 illustrates the workflow of an integrative clustering analysis. The iCluster method simultaneously achieves data integration and dimension reduction through a joint latent variable model. The goal is to identify a set of driving factors that define biologically and clinically relevant subtypes of the disease. This is best explained by an example. In the well-known *HER2* breast tumor subtype, the driving characteristic of the subtype is the *HER2* oncogene activation through concordant DNA amplification and mRNA overexpression of genes within the *HER2* amplicon (Supplementary Figure S1D). Based on existing knowledge on the driving factors (*HER2* in this case) in a certain cancer type and the observed data for each tumor, we can model the patients' multidimensional genomic profile as functions of the driving factors for effective data integration and dimension reduction. However, in the general problem of class discovery, the driving factors are not known and need to be identified from the multidimensional data space. This motivates us to consider a latent variable modeling framework.

**Figure 1:**
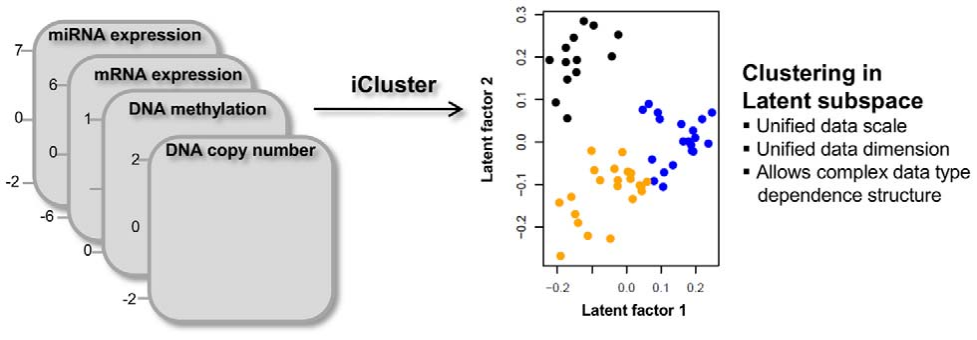
The iCluster framework.

The model induces complex dependence structures among different genomic data types using latent variables that represent the underlying cancer driving factors. Integration and dimension reduction is achieved through simultaneous projection of the multidimensional data space of different dimensions and scales onto a lower dimensional latent subspace of unified dimension and scale (Figure 1). The resulting latent subspace reveals cluster structures among the sample points. The coefficient matrix determines the relationship between the original features and the latent variable. A variance-weighted adaptive shrinkage method is applied to impose sparsity on the loading matrix (many entries are zero) for selecting cluster-discriminant features as part of the iCluster procedure. Revisiting the *HER2* subtype example, the loading vector associated with the *HER2* subtype will only have nonzero values for genes within the *HER2* amplicon and zeros everywhere else (Supplementary Figure S1F).

### Comparison to separate clustering and naive integration using simulation

Separate clustering followed by manual integration remains the most frequently applied approach to analyze multiple omics data sets in the current literature for its simplicity and the lack of a truly integrative approach. Using simulation analysis, we demonstrate that separate clustering can fail drastically in estimating the true number of clusters, classifying samples to the correct clusters, and selecting cluster-associated features. In Table 1, the simpler method (separate K-means) chooses the correct number of clusters (k) only 60% of the time with an average cluster reproducibility of 0.68. By contrast, iCluster estimates the correct number of clusters 90% of the time with an average cluster reproducibility of 0.81. In a second simulation scenario with a more sparse data structure in which only two features are relevant to define the clusters, the iCluster method outperforms the competing approach by a substantial margin in terms of the ability to choose the correct number of clusters (40% vs 92% accurate), cross-validated error rates (0.11 vs 0.01), and cluster reproducibility (0.48 vs 0.98) (Simulation Scenario 2 in Table 1). This simulation analysis indicates that care should be taken in the current standard practice when interpreting results from separate clustering of multidimensional data sets.

**Table 1:** Comparing separate clustering and iCluster performance using simulation.

	Simulated Scenario 1		
Method	Freq estimating the correct K	Error Rate	RI
Separate K-means	60%	0.08 (0.04)	0.68 (0.18)
Sparse iCluster	90%	0.04 (0.02)	0.81 (0.08)

	Simulated Scenario 2		
Separate K-means	40%	0.11 (0.06)	0.48 (0.20)
Sparse iCluster	92%	0.01 (0.01)	0.98 (0.04)

Simulation scenario 1 consists of a pair of matched data sets of 200 features (20 of which are relevant to clustering and 180 are noisy features) in 100 samples belonging to two distinct clusters. Scenario 2 represents an extremely sparse data structure with only 2 cluster-associated features and 198 noisy features. RI is the resampling-based cluster reproducibility criterion and ranges between 0 and 1. A value close to 1 indicates perfect cluster reproducibility, and a value close to 0 indicates poor reproducibility. Separate K-means has two sets of numbers associated with each criterion because of separate model fits. The numbers are similar and therefore averaged in the table. The number in parentheses is the standard deviation over 50 simulations.

**Figure 2:**
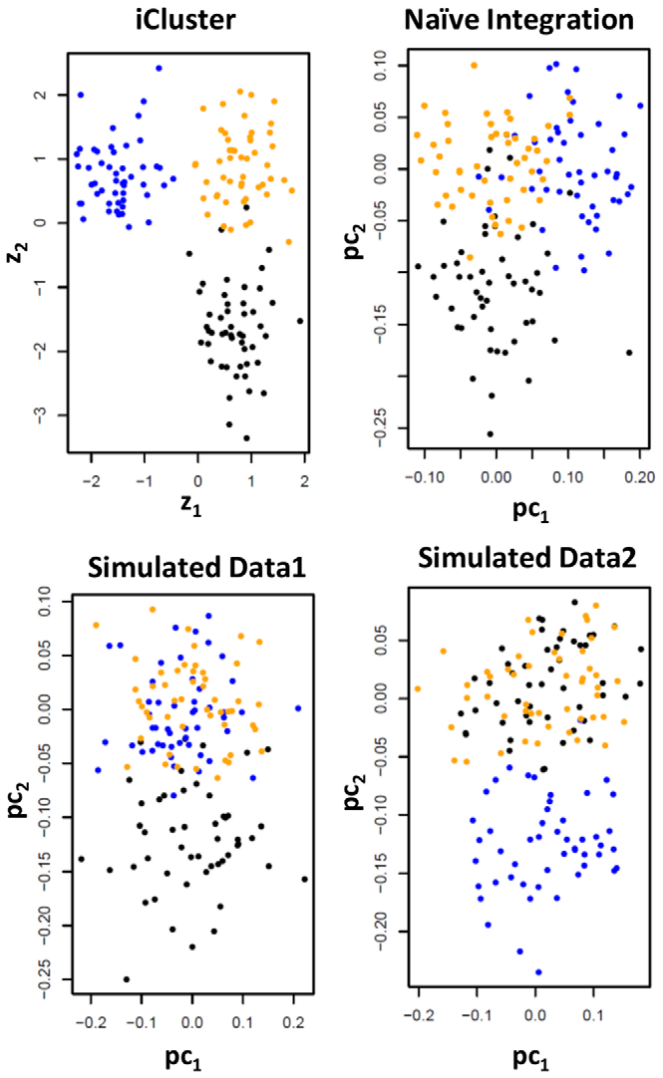
Comparing iCluster to a naive integration via PCA using simulated data. Two-dimensional plots of the sample points in the latent subspace by different methods. A set of 150 subjects are simulated belonging to three clusters (indicated by black, blue, and orange dots). Each subject has a pair of synthetic molecular profiles representing two data types each consisting of 1,000 features. A common set of 5 correlated features in both data type 1 and 2 defines the black subtype. Another set of 5 features specific to data type 2 defines the blue subtype. The remaining features are noise.

We also compared iCluster to a principal component analysis (PCA) based integration. PCA is known to reveal cluster structures in a lower dimensional latent subspace. Given multiple data types, we applied PCA to the combined data matrices. Using simulated data, Figure 2 shows that such naive integration cannot separate the subgroups with high accuracy. By contrast, iCluster clearly outperforms the naive integration as well as the separate clusterings in discriminating the true clusters.

### iCluster Identifies Three Distinct Molecular Subtypes of Glioblastoma

In recent TCGA publications on glioblastoma subtypes, Verhaak et al. (2010) [19] identified four distinct expression subtypes: Proneural, Neural, Classical and Mesenchymal using 1,740 most variable genes. In addition, Noushmehr et al. (2010) [20] reported a Glioma-CpG Island Methylator Phenotype (G-CIMP) based on 1,503 methylation features. We hypothesize that an integrative subtype analysis would be a more powerful approach to characterize subtypes with coordinated genomic, epigenomic, and transcriptomic alterations. To that end, we applied the iCluster algorithm for a joint analysis of copy number, methylation and gene expression on a subset of 55 glioblastoma samples (see Data Set Section).

**Figure 3:**
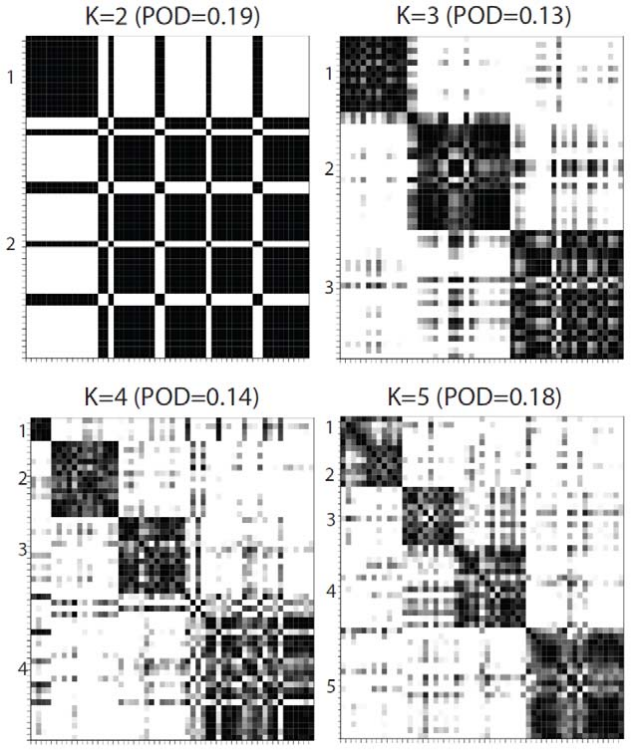
Cluster separability plots in the GBM data set. Proportion of deviation (POD) is calculated as the proportion of deviation from a block diagonal structure. K = 3 has the best block-diagonal structure.

**Figure 4:**
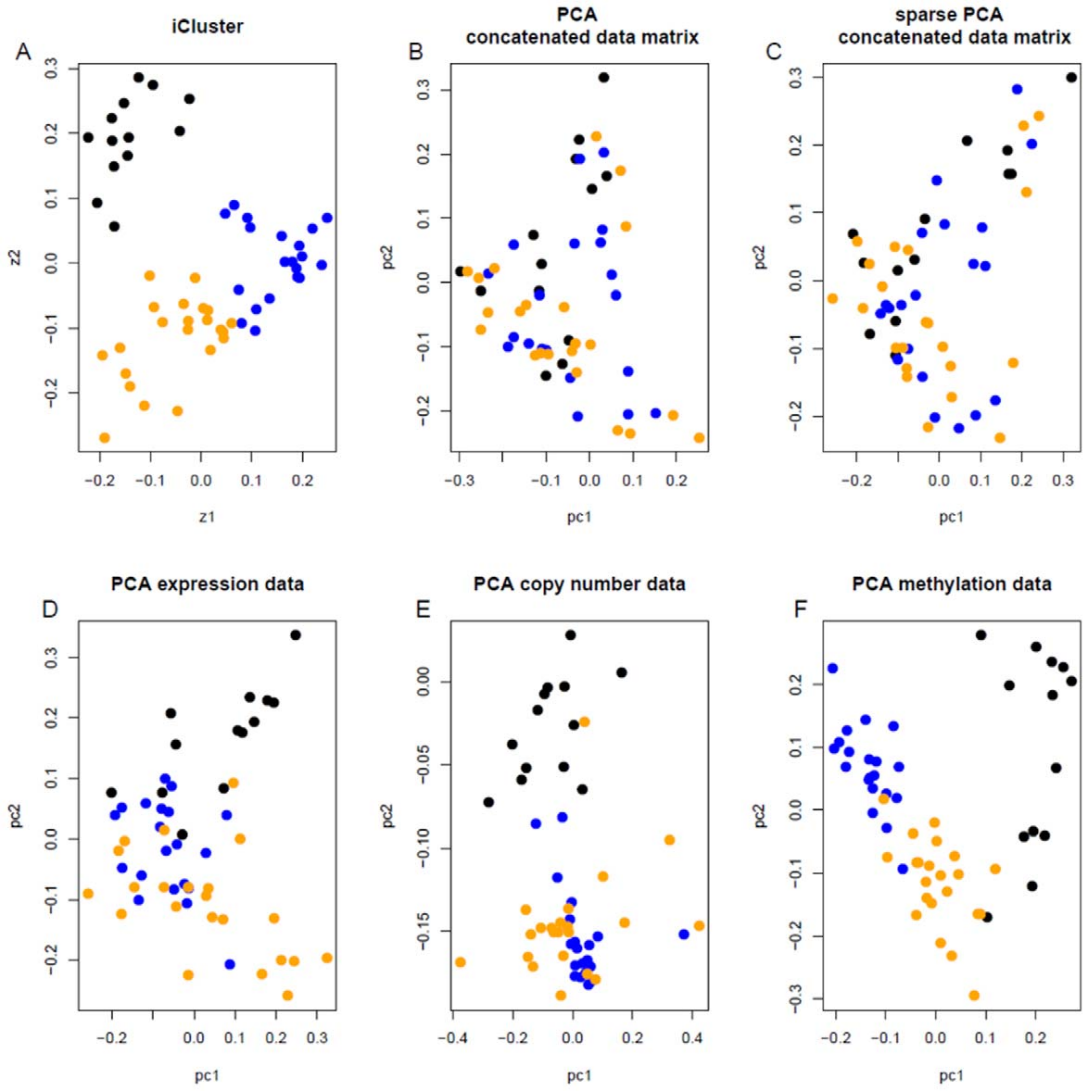
Comparison of the iCluster method to PCA approaches in the GBM data set. Two-dimensional plots of the sample points in the latent subspace spanned by A) the first two joint latent factors obtained using iCluster, B) the first two principal components (PCs) from the concatenated data matrix, C) the first two sparse PCs from the concatenated data matrix, D) the first two PCs from the mRNA expression data alone, E) from the copy number data alone, and F) from the methylation data alone.

The number of reproducible subtypes (K) and model sparsity (number of subtype-discriminant features) are determined using a resampling-based scheme as described in the Methods Section. Within each iteration of the algorithm, a reproducibility index (RI) is computed for each point drawn from the parameter space based on a uniform sampling design. An RI close to 1 indicates perfect cluster reproducibility and an RI close to 0 indicates poor reproducibility. Table 2 indicates highly reproducible solutions by integrative clustering. Both K = 2 and K = 3 are highly reproducible with RI = 1.00 and 0.93 respectively. We further examine the cluster separability plots (see Methods) in Figure 3, which reveals that K = 3 gives the best diagonal block structure. Overall, we find combining the cluster reproducibility and separability measure is an effective way for choosing the number of clusters given complex data structures. In Figure 4, iCluster outperforms the competing methods in revealing subgroup structure in the lower dimension latent subspace. The standard PCA and a sparse PCA approach [9] applied to the concatenated data matrix did not achieve satisfactory results (Figure 4B and 4C).

**Table 2:** Integrated subtype reproducibility and number of subtype-discriminant features.

Reproducibility Index	CN features	Methyl features	Exp features
1.00	104	74	91
0.93	308	240	228
0.54	713	272	285
0.63	550	631	488
0.41	453	672	237

**Figure 5:**
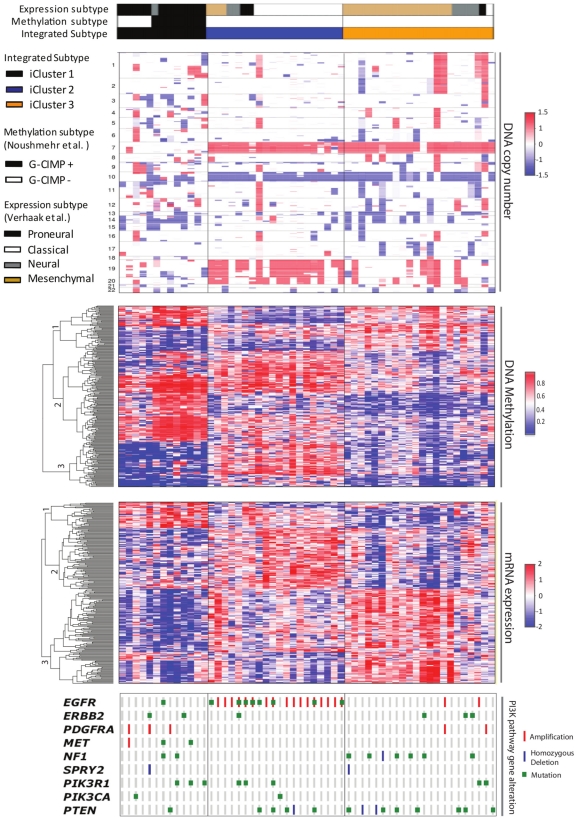
iCluster reveals three distinct glioblastoma integrated subtypes. iCluster was applied using 1,599 copy number features, 1,515 DNA methylation features, and 1,740 expression features. Heatmap display of the subset of cluster-discriminant features reveals highly coordinated pattern of alteration in copy number, methylation, and expression. Integrated subtype 1 shows a “sporadic” profile of copy number alterations; hypermethylation of genes involved in brain development and neuronal differentiation, and a proneural expression profile. Integrated subtype 2 is characterized by a near complete association with EGFR alteration, gains of chr 19 and 20, methylation of homeobox genes, and a classical-enriched expression profile. Integrated subtype 3 is characterized by NF1 and PTEN alterations and exhibits mesenchymal-like expression. The TCGA expression subtype and the G-CIMP subtype memberships are aligned on top of the integrated subtype membership as color-coded labels. PI3K pathway activity is shown at the bottom of the figure.

**Figure 6:**
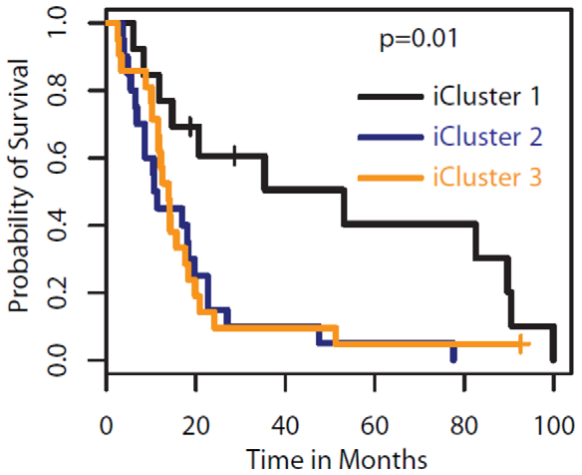
Kaplan-Meier plot. The three integrated subtypes of glioblastoma identified by iCluster show survival differences.

Figure 5 reveals the major characteristics for each of the three integrated GBM subtypes. The most notable feature of the Glioblastoma subtype 1 identified by iCluster is the lack of chr7 gain and chr10 loss (the classical GBM events), and shows a “sporadic” profile of copy number alterations. This subclass is enriched for the G-CIMP phenotype and shows hypermethylation of genes involved in brain development and neuronal differentiation including *DLC1*, *JAG2*, *and ALDH1A3* (Supplementary Table S1). The expression phenotype of the tumors in this subclass is predominantly Proneural. This subclass of patients show significantly better survival (P = 0.01) than the other two clusters (Figure 6). Subtype 2 is characterized by a near complete association with *EGFR* amplification, gains of chr 19 and 20, methylation of homeobox genes including *IRX2* and *BARHL2* and G-protein signaling genes including *CXCL6* and *DRD5*, and a classical-enriched expression profile. Subtype 3 is characterized by *NF1* and *PTEN* alterations and exhibits mesenchymal-like expression.

### Joint feature selection reveals coordinated genomic and epigenomic regulation

As mentioned earlier, feature selection is an integral part of the iCluster algorithm and is accomplished via an adaptive shrinkage estimation of the coefficient matrix. A genomic feature is associated with a subtype if the corresponding shrinkage-based coefficient (factor lading) estimate is nonzero. As a result, clustering variability can be substantially reduced by effectively removing noninformative features by forcing their coefficients to zero. As mentioned earlier, Table 1 clearly shows that the sparse models, as a result, lead to significantly better cluster reproducibility than their nonsparse counterparts. The performance of the latter using all features is degraded by noise accumulation. The full lists of selected features arranged in the corresponding gene cluster can be found in Supplementary Tables S1 and S2.

**Figure 7:**
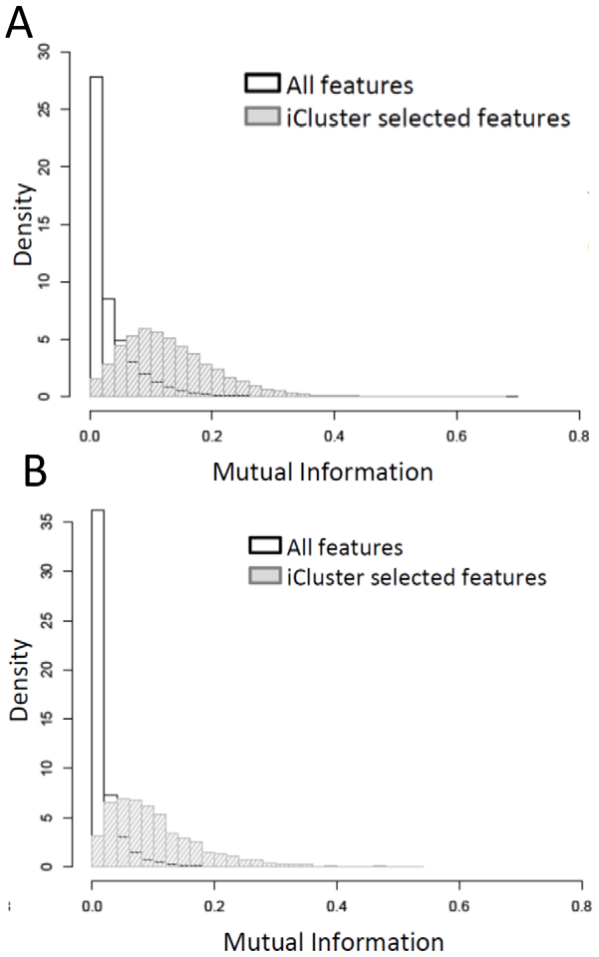
Mutual information.

Mutual information I(X,Y) is a measure of dependence between two random variables that is considered more general and robust than correlation. It is a nonnegative measure with I(X,Y) = 0 indicating independence. [21] used mutual information to quantify on a global level the extent to which entropy increase in one random variable (DNA copy number) leads to an entropy increase in another (gene expression). Figure 7 shows the distribution of all pair-wise mutual information between DNA methylation and gene expression (7A), and between DNA copy number and gene expression (7B). The unselected feature space (all features) is dominated by features with low mutual information content, whereas the iCluster selected feature space is substantially enriched for features with high mutual information content with the distribution considerably shifted to the right.

## Discussion

Integrative genomic studies given multiple omic dimensions carry the promise of more power to characterize, classify, and predict outcomes in cancer than the conventional genomic study involving gene expression data alone. We present a unified data analysis framework that conducts clustering, data integration, feature selection, and dimension reduction simultaneously to harness the full potential of large-scale integrated cancer genomic data. As we illustrated using the TCGA GBM data set, a strength of an integrative clustering analysis is the ability to discover and visualize coordinated patterns of genomic alterations, providing a biologically comprehensive context for subtype discovery.

A practical challenge for validating integrated clusters is the availability of independent data sets with all data types available. With the accumulating number of integrated genomic profiling studies, we expect this problem will become less severe over time. In the GBM data set, each data type shows distinct cluster-discriminating patterns (Figure 5). A natural question then arises as to what degree a single data type (e.g., copy number) can reproduce the integrated subtype label generated by iCluster. To that end, we conducted an internal cross-validation (CV) based on the copy number profile alone using a k-nearest neighbor method. In leave-one-out cross-validation, we find the k nearest neighbors of the left-out sample (based on the Euclidean distance of the copy number profiles) and then classify the left-out sample to the corresponding class label with the majority votes. We iterate this procedure until all the samples are left out once and assigned a corresponding class label. The CV error rate is then computed as the percentage of misclassified subtype memberships. The k chosen is the one that minimizes this CV error rate. The procedure can be similarly applied to other data types. Using this internal cross-validation procedure, we found using copy number data alone could assign 77% of the samples to the correct integrated subtype. Through a similar procedure, we obtained an 87% accuracy using the expression data alone, and a 93% accuracy using the methylation data alone for classifying the samples to the correct integrated subtype label.

Given the reasonably good cross-validation performance, we then applied this single data type validation approach using an independent set of 136 samples from the same TCGA GBM cohort that were not included in the integrative clustering analysis for reasons discussed in Materials and Methods. We assigned cluster membership for each of the 136 samples based on the majority voting of the k-nearest neighbor approach based on copy number profiles alone (Supplementary Figure S2) and based on their gene expression profile alone (Supplementary Figure S3). Both clearly indicate that the distinct copy number and gene expression patterns in Figure 5 can be validated in the independent sample set.

In general practice, a validation study requires the availability of all data types used for the discovery of the integrated subtypes. However, as we have shown here, an internal cross-validation can be used to assess the degree to which each single data type alone can reproduce the integrated cluster membership. If a single data type can replicate the integrated subtypes with sufficient accuracy, then it may not be necessary to collect all of the data types in subsequent validation experiments.

## Materials and Methods

### Data set

A description of the TCGA data types, platforms and analyses can be found in TCGA (2008). The GBM data set were downloaded from the Cancer Genome Atlas public data portal, and from the cBio Cancer Genomics Portal (http://cbioportal.org/) at the Memorial Sloan-Kettering Cancer Center. For copy number data (n = 206), “level 3” normalized and segmented data from Agilent 244 K CGH arrays were used. In a typically data pre-processing step, we use CGHregion [22] to reduce multi-sample array CGH data to 1 K–5 K unique regions. In this study, however, we use the gene-centric data generated using RAE [23] to facilitate interpretation and comparison with published results. For mRNA expression data (n = 202), unified gene expression data across three microarray platforms (Affymetrix Human Exon 1.0 ST GeneChips, Affymetrix HT-HG-U133A GeneChips, and custom designed Agilent 244 K array) as described in [19] were used. A final set of 1,740 most variable genes were used for the analysis. The GBM methylation data were generated on two different platforms: Illumina Infinium and GoldenGate. We used the higher resolution data from the Infinium 27 K platform in this study (n = 91). A set of 1,515 most variable probes (beta values) were used for the analysis. The final “triplet” dataset for integrative analysis (copy number, expression, methylation) consists of a total of 55 samples where all three data types as described above are available.

### Sparse joint latent variable model

Suppose 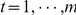 different genome-scale data types (DNA copy number, methylation, mRNA expression, etc.) are obtained in 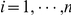 tumors. Let 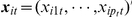 denote a 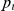-dimensional genomic data vector. Each element 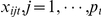, represents the observation associated with the 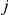
th genomic feature of type 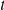 measured in tumor 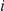. To facilitate the discussion of feature selection in this paper, we use *genomic feature* as a general term to refer to protein-coding genes as well as non-coding genetic and genomic elements depending on the platform and data type. The details of the integrative clustering method can be found in [17], [24]. Briefly, the sets of 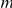 genomic data vectors 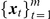 are related to a common (shared) set of latent variables 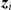 using the following model
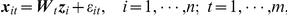
where 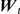 denotes the coefficient (loading) matrix associated with data type 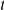, and 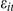 denotes the error term with mean zero and a diagonal covariance matrix 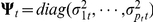, representing the residual variance.

The iCluster framework simultaneously achieves data integration and dimension reduction. The concept of the model is depicted in Figure 1 and Supplementary Figure S1. The common latent variable vector 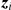 represents the underlying driving factors in tumor 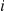 that can be used for disease subtype assignment. It is also a key instrument for inducing complex dependence structures between data types and as a result renders an effective integration scheme across multiple correlated data sources. At the same time, dimension reduction is achieved through projecting the multidimensional data space to a low dimensional integrated subspace: 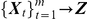, where 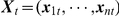 is the data matrix of dimension 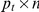 and 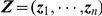 is the latent factor matrix of dimension 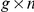. Typically, 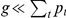, providing a low-rank joint approximation to the original data sets. We assume a rank-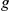 approximation where 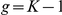 for separating 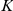 clusters among the 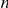 data points.

### Parameter Estimation

The EM algorithm [25] is used for parameter estimation. Given Gaussian error terms 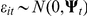, the Expectation step (E-step) entails computing the posterior mean and variance of the latent factors, and the Maximization step (M-step) leads to estimates of the coefficient matrix and the error covariance matrix. The algorithm iterates between E-step and M-step until convergence.

Sparsity in the estimate of 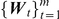 are important for balancing between model fit and model complexity. In the original paper [17], we proposed to use a lasso approach that penalizes the 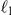 norm of the coefficient vectors and continuously shrinks the coefficients associated with noninformative genes toward zero. Let 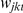 denote the element in row 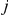 and column 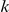 of the coefficient matrix 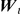, we considered the following shrinkage estimates:
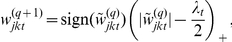
where 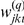 is the standard maximum likelihood estimates at the 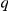-th EM iteration, and 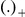 denotes the positive part. When the penalty parameter 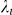 is sufficiently large, many of the coefficient estimates will be exactly zero. If 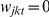, feature 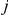 in data type 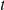 has has no bearing on the 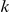
th latent factor. A sparse 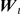 with lots of zero elements is more interpretable and provides a framework for selecting cluster-discriminant features.

In this paper, we consider an adaptive-type penalty that is proportional to the variance of each feature in the following form:
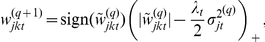
where the shrinkage term is proportional to the variance 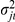 associated with genomic feature 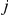 in data type 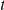. Coefficients will be more heavily penalized for features demonstrating high variance.

### Choice of Tuning Parameters

We use a cluster reproducibility index (RI) as described in [24] for choosing the number of clusters (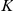) and the degree of sparsity (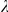) in the genomic feature space. It entails repeatedly partitioning the samples into a learning and a test set and evaluating the degree of agreement between the predicted and the fitted (“observed”) cluster assignment using an adjusted Rand index. The procedure is depicted in Supplementary Figure S1. Values of RI close to 1 indicate perfect cluster reproducibility and values of RI close to 0 indicate poor cluster reproducibility. In this framework, the concept of prediction error that typically applies to classification analysis where the true cluster labels are known now becomes relevant for clustering [26]–[28].

For visualization of the sample similarity matrix, Shen et al. (2009) [17] described a cluster separability plot based on the product matrix of the posterior mean of the latent factors. Perfect cluster separability (non-overlapping clusters) would lead to an exact diagonal block matrix with diagonal blocks of ones for samples belonging to the same cluster and off-diagonal blocks of zeros for samples in different clusters. The corresponding proportion of deviance (POD) measure is between 0 and 1. Small values of POD indicate strong cluster separability, and large values of POD indicate poor cluster separability.

### Sampling design

In the integrative space, an exhaustive grid search for the optimal combination of (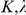) that maximizes cluster reproducibility is inefficient and computationally prohibitive. To overcome this obstacle, we use the uniform sampling design (UD) approach of Fang and Wang (1994) [29] to generate experimental points that scattered uniformly across the search domain. It has been shown that UD has superior convergence rate than the traditional grid search over the parameter space [29]. Suppose we apply iCluster on two data types (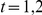) with a parameter tuning process that involves finding the best values for (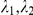), the sparsity-inducing penalty parameters as described earlier. Each of the penalty parameters ranges between 0 and 1, with 0 representing the null model where no features are selected and 1 representing the full model where all features are included. Supplementary Figure S4 shows an example of the UD sampling pattern where 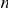 here denotes the number of ‘trials’ in which we fit the iCluster model with the chosen combinations of (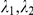) uniformly sampled from the search domain 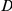. A key theoretic advantage of the uniform design over the traditional grid search is the uniform space filling property that avoids wasteful computation at close-by points. As we can see in Supplementary Figure S4, each value of (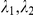) only appears once in the UD design, an important characteristic for efficient model selection. The parameter points used to generate UD sampling patterns are chosen by number-theoretic methods (Fang and Wang,1994) that achieve uniform and space-filling properties. The UD tables can be found at the following link: http://www.math.hkbu.edu.hk/UniformDesign/.

### Mutual information

Mutual information is a general measure of certain functional dependence (unrestricted to linear dependence) between two random variables. van Wieringen and van der Vaart (2011) [22] uses mutual information to quantify the extent to which entropy increase in one random variable (DNA copy number) leads to an entropy increase in another (gene expression). The classic definition of mutual information between two random variable is
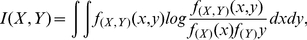
(1)where 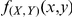 is the joint density function of 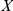 and 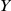, and 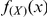 and 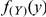 are the marginal density functions. Mutual information of two Gaussian random variable is known to be 
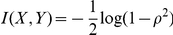
 where 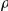 is the correlation which is what we used in Figure 7.

## Supporting Information

Figure S1 **Integrative Clustering Analysis Workflow.**
(PDF)

Figure S2 **Validation using copy number data alone.**
(PDF)

Figure S3 **Validation using gene expression data alone.**
(PDF)

Figure S4 **Two-dimensional uniform sampling.**
(PDF)

Table S1 **Selected methylation features and functional annotations using DAVID.**
(XLSX)

Table S2 **Selected expression features and functional annotations using DAVID.**
(XLSX)
