# Supplementary figures and images for: Integrative Subtype Discovery in Glioblastoma Using iCluster
Source: PLoS One. 2012 Apr 23;7(4):e35236. doi: 10.1371/journal.pone.0035236 (PMC3335101; doi:10.1371/journal.pone.0035236)

# Supplementary Figure 1

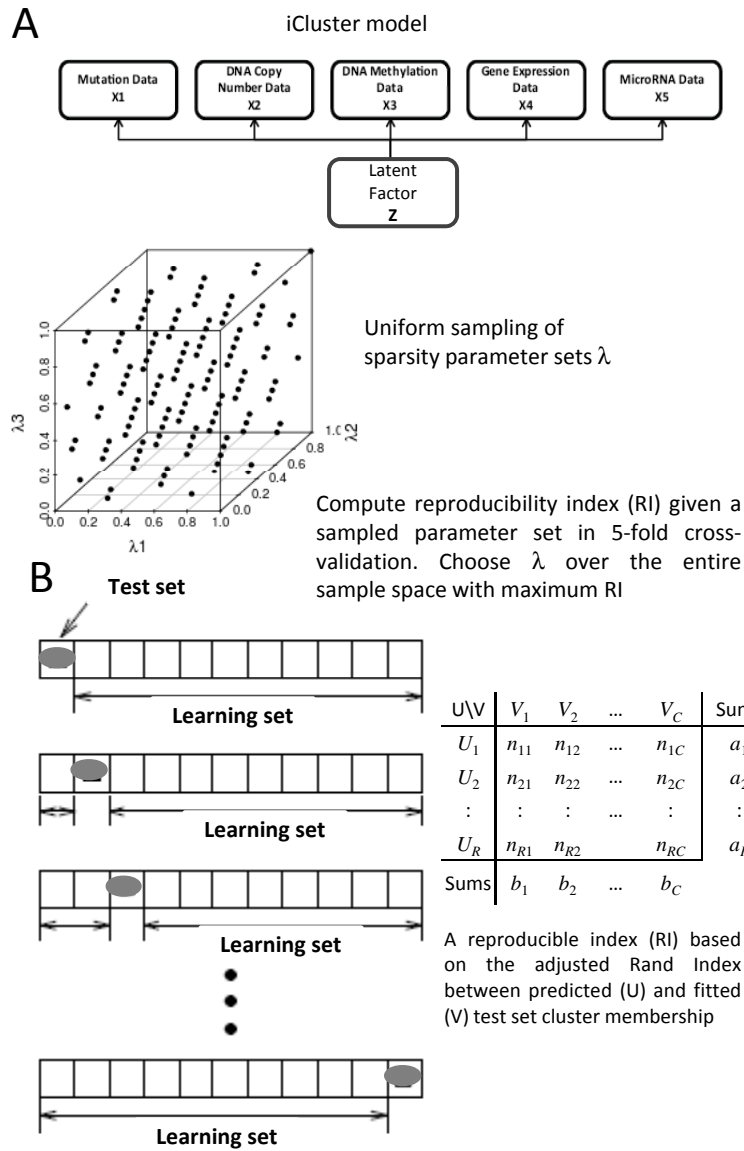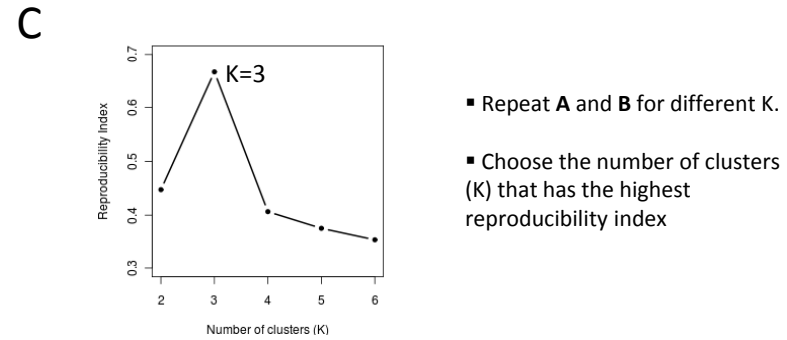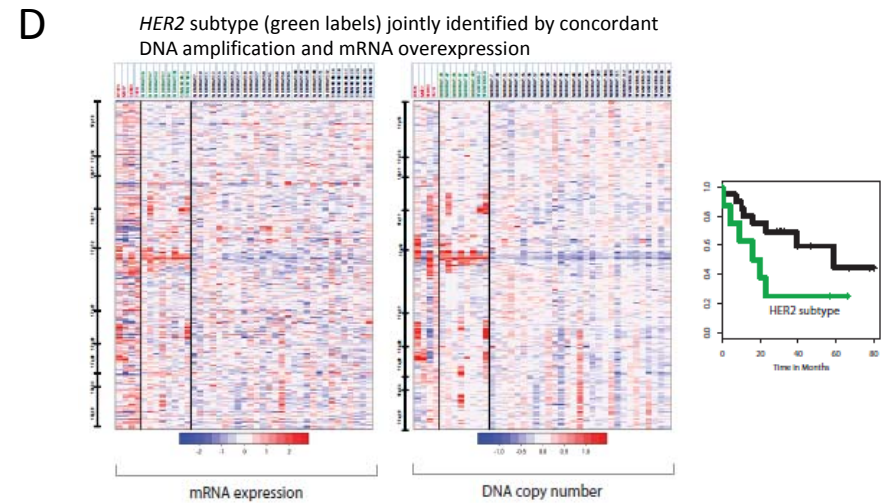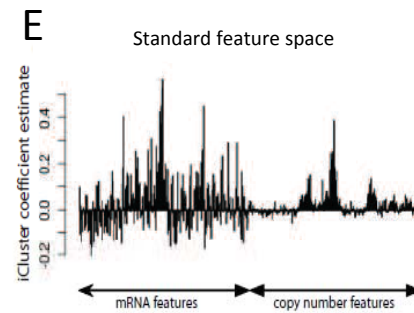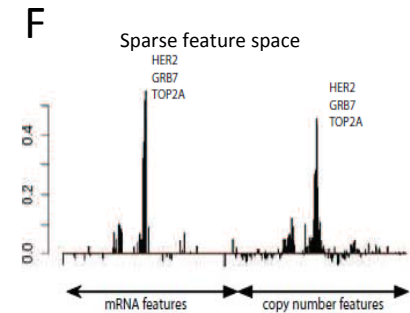

Supplement: Figure S1 — Integrative Clustering Analysis Workflow. (PDF) [file pone.0035236.s001.pdf]

Supplementary Figure 2

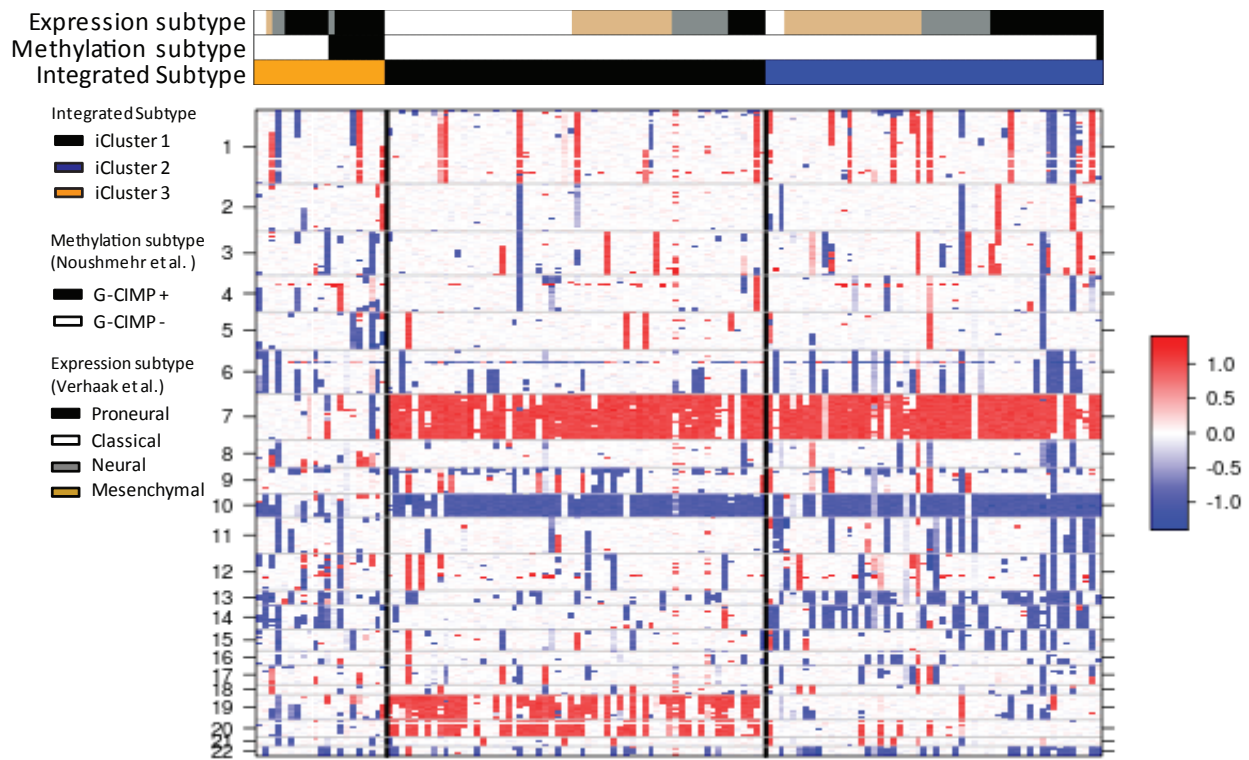

Supplement: Figure S2 — Validation using copy number data alone. (PDF) [file pone.0035236.s002.pdf]

Supplementary Figure 3

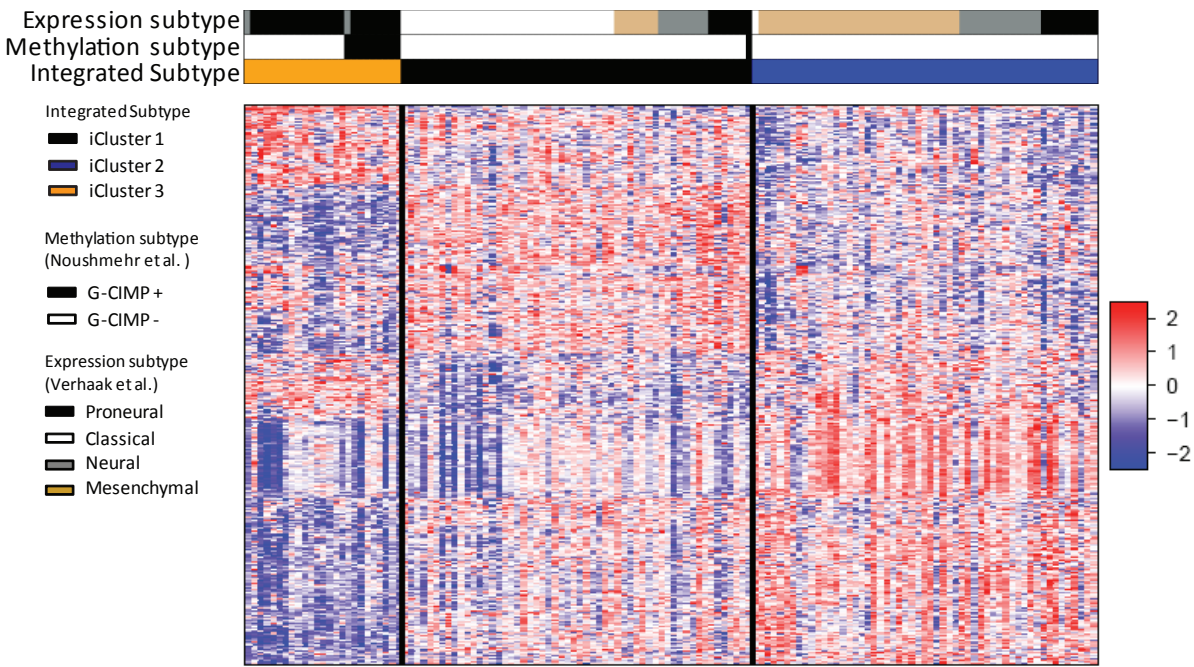

Supplement: Figure S3 — Validation using gene expression data alone. (PDF) [file pone.0035236.s003.pdf]

Supplementary Figure 4

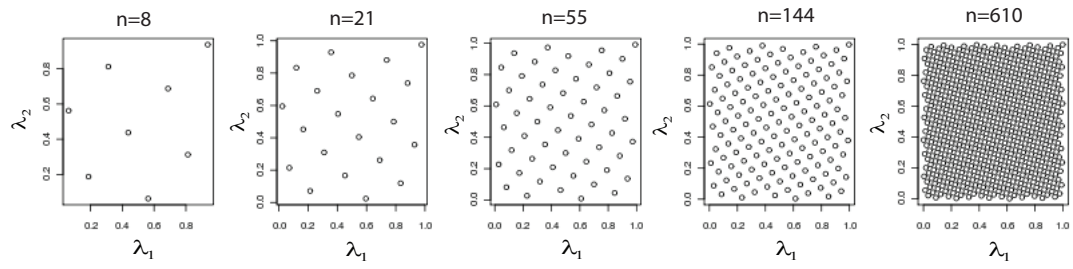

Supplement: Figure S4 — Two-dimensional uniform sampling. (PDF) [file pone.0035236.s004.pdf]
